# Protection Patterns in Duck and Chicken after Homo- or Hetero-Subtypic Reinfections with H5 and H7 Low Pathogenicity Avian Influenza Viruses: A Comparative Study

**DOI:** 10.1371/journal.pone.0105189

**Published:** 2014-08-25

**Authors:** Coralie Chaise, Anne-Christine Lalmanach, Hélène Marty, Sébastien Mathieu Soubies, Guillaume Croville, Josyane Loupias, Daniel Marc, Pascale Quéré, Jean-Luc Guérin

**Affiliations:** 1 Universite de Toulouse, Institut National Polytechnique de Toulouse (INPT), Ecole Nationale Vétérinaire de Toulouse (ENVT), Toulouse, France; 2 Institut National de la Recherche Agronomique (INRA), Unité Mixte de Recherche 1225 Interactions Hôtes – Agents Pathogènes (IHAP), Toulouse, France; 3 Institut National de la Recherche Agronomique (INRA), Unité Mixte de Recherche 1282 Infectiologie et Santé Publique (ISP), Nouzilly, France; 4 Université François Rabelais, Tours, France; The University of Chicago, United States of America

## Abstract

Avian influenza viruses are circulating continuously in ducks, inducing a mostly asymptomatic infection, while chickens are accidental hosts highly susceptible to respiratory disease. This discrepancy might be due to a different host response to the virus between these two bird species and in particular to a different susceptibility to reinfection. In an attempt to address this question, we analyzed, in ducks and in chickens, the viral load in infected tissues and the humoral immune response after experimental primary and secondary challenge infections with either homologous or heterologous low pathogenicity avian influenza viruses (LPAIV). Following homologous reinfection, ducks were only partially protected against viral shedding in the lower intestine in conjunction with a moderate antibody response, whereas chickens were totally protected against viral shedding in the upper respiratory airways and developed a stronger antibody response. On the contrary, heterologous reinfection was not followed by a reduced viral excretion in the upper airways of chickens, while ducks were still partially protected from intestinal excretion of the virus, with no correlation to the antibody response. Our comparative study provides a comprehensive demonstration of the variation of viral tropism and control of the host humoral response to LPAIV between two different bird species with different degrees of susceptibility to avian influenza.

## Introduction

Anseriformes and Charadriiformes are considered as the main reservoir of Low Pathogenicity Avian Influenza Viruses (LPAIVs) (reviewed in [1]). Among aquatic birds, mallards (*Anas platyrhynchos*) and their domestic counterparts Pekin ducks (*Anas platyrhynchos domesticus*), are of particular epidemiological importance [2]. In ducks, LPAIV infections are mainly asymptomatic and restricted to the epithelial cells lining the distal digestive tract [3]. The subsequent release of virus in feces is therefore a main source of contamination for the environment [4]. Prevalence in wild and domestic waterfowl seems high, irrespective of viral subtype. Factors and mechanisms underlying the active circulation of LPAIVs in ducks populations remain to be determined. Immunity generated by a primary infection against a reinfection is likely to play a critical role in the host capacity to control viral persistence at the population level. However, this remains poorly documented.

In gallinaceous birds, LPAIVs are far less prevalent, and are mainly replicating in the respiratory tract, leading to moderate to severe clinical signs [2]. Several studies have investigated the adaptive immunity following infection with homologous or heterologous AIV infections [5], [6]. Most of these studies addressed protection against infection with H5N1 highly pathogenic avian influenza viruses (HPAIV) [7], [8]. Humoral immune response is known to play an important role in the control of avian influenza virus infections by reducing virus replication and spread [9].

Aquatic birds and terrestrial fowl respond differently to the viral infection, which suggests that they could mount a different immune response to infection, owing to the specificity of their respective antiviral immunity. This intriguing question led us to compare ducks and chickens for the protection against a LPAIV infection conferred by a primo-infection, either by the same strain (homologous reinfection) or by a different subtype (heterologous reinfection). The level of protection was investigated in each host species using a similar experimental LPAIV infection protocol for primo- and reinfection and by measuring the viral load and the antibody response shown to be a major mechanism involved in protection to a secondary influenza infection.

## Materials and Methods

### Animals

One-day-old female Pekin ducks (*Anas platyrhynchos domesticus*) were purchased from a commercial hatchery of controlled sanitary status (Couvoir de la Seigneurtière, Vieillevigne, France). White Leghorn chickens, histocompatible for the B13 haplotype (GB1 Athens chicken line), were hatched and raised free of specific pathogens at INRA (Platform for Experimental Infectiology, Nouzilly, France) until 3 weeks of age.

Birds were housed in BSL3 poultry isolator units for infection experiments. Food and water were provided *ad libitum*. All animals used in these experiments were treated according to EU recommendations for animal welfare and the protocol was approved by the Indre et Loire (37, France) French Ethics Committee (#2010/3), in strict compliance with legal dispositions applicable in France until 1^st^ of February 2013 (French Government Decree 2001-464 of 29 May 2001) and under veterinary surveillance. Experiments were performed in facilities under authorization and supervision of official veterinary services (authorization # C3155527 delivered the 19^th^ of October, 2010).

In order to control the status of birds before AIV exposure, blood, cloacal and oropharyngeal swab samples were collected from 25 ducks prior to inoculation. All birds were found to be seronegative for influenza A antibodies as determined by ELISA (see procedure in the serology subsection below); influenza A-specific real-time RT-PCR was also negative for all animals (see procedure in the virus titration subsection below). In order to verify their “specific pathogen-free” (SPF) status, the same tests were performed on a few randomly chosen chickens, and found negative.

### Viruses

The two low pathogenicity avian influenza (LPAI) viruses A/Duck/Italy/775/2004 (H5N3) (NCBI Taxon ID: 437394) and A/Duck/Italy/4609/2003 (H7N2) (NCBI Taxon ID: 475510), were kindly provided by Dr Ilaria Capua (IZPS Legnaro, Padova). The H5N3 virus (hereafter named “H5”) was used for the primary infections, while reinfection experiments were performed either with the same H5 virus, or with the H7N2 virus (hereafter named “H7”). Working stocks of both viruses were propagated and titrated in 9-day-old SPF embryonated chicken eggs (ECE), as previously described [10].

### Experimental infections of ducks and chickens

**Figure 1:**
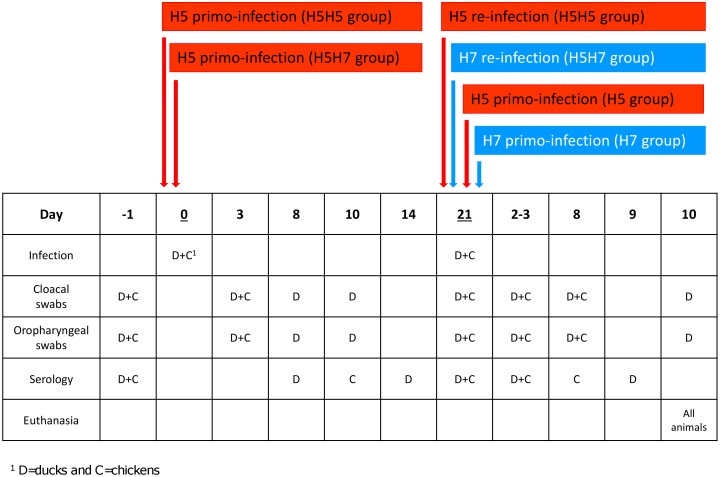
Design of experimental infections of ducks and chickens. ^1^animals were primo-infected by intranasal and oropharyngeal routes with 4.10^6^ PFU of H5N3 virus, ^2^animals were primo or reinfected with 4.10^6^ PFU of H5N3 (in red) or H7N2 viruses (in blue), ^3^OS =  Oropharyngeal Swabs, ^4^CS =  Cloacal Swabs, ^5^Sero =  Serology.

A similar experimental design was applied to chickens and ducks, following previously described procedures [11]. For animal experiment on Pekin ducks, 15 birds were inoculated at three weeks of age *via* intranasal and oropharyngeal routes with 4×10^6^ plaque-forming units (PFU) of the H5 virus. As described in figure 1, cloacal (CS) and/or oropharyngeal (OS) swabs were collected from each duck at 3, 8, 10 and 21 days post-inoculation (p.i.) and stored at −80°C until their evaluation through influenza A-specific real-time RT-PCR. In addition, sera were collected from all ducks at 8, 14 and 21 days post-inoculation (Figure 1) and were stored at −20°C until their evaluation through ELISA. Twenty-one days p.i. (i.e. at 6 weeks of age), infected ducks were divided into two groups (Figure 1). One group was challenged *via* intranasal and oropharyngeal routes with 4×10^6^ PFU of the same H5 virus (H5H5 group; n = 10), while the second group was challenged *via* the same routes with 4×10^6^ PFU of the H7 virus (H5H7 group; n = 5). In parallel, two naive groups were primo-inoculated at 6 weeks of age in the same way with the same dose of either the H5 virus (H5 group; n = 5) or the H7 virus (H7 group; n = 5). The four groups were subsequently housed separately. Cloacal and oral swabs were collected at 3, 8 and 10 days p.i from each duck of the 4 groups for virus isolation. In addition, blood was collected from all ducks at 3 and 9 days p.i. to test for antibodies against the H5 or H7 viruses (Figure 1). All ducks were euthanized at 10 days after challenge by intravenous administration of sodium pentobarbital (Merial, France). For experimental infections of chickens, 28 birds were divided into four groups of 7 animals. Birds in the first two groups were primo-infected at 3 weeks of age with the H5 virus, using the same protocol as that used in ducks (see figure 1). They were then kept in two separate isolators. Birds in the third and fourth groups were kept uninfected (naive groups). OS and CS were sampled from chickens of the different groups at 3 days p.i. and processed in the same way as for ducks. In addition, blood was collected from chickens of all groups at 10 days p.i. Three weeks after the first inoculation (i.e. at 6 weeks of age), primo-infected birds were inoculated with either the H5 (H5H5 group, n = 7) or the H7 virus (H5H7 group, n = 7), following the same procedure as for ducks. The two naïve groups were challenged identically (H5 group, n = 7; H7 group, n = 7). At day2 and 8 post-challenge, cloacal and oral swabs were collected from seven chickens from each group for virus isolation, as well as sera from all chickens to test for H5N3- or H7N2-specific antibodies. Chickens were then euthanized 10 days post-challenge by intravenous injection of sodium pentobarbital (Merial, France).

### Serology

All blood samples were collected from the saphenous vein for ducks or from the wing vein for chickens. Serum samples were stored at −20°C until being tested for the presence of influenza A antibodies using an enzyme-linked immunosorbent assay (ELISA) or Hemagglutination Inhibition test (HI).

For ELISA tests, the H5 and H7 viruses were amplified in MDCK cells, the supernatant was clarified (3000×g, 30 min), then the virus was pelleted by ultracentrifugation (100 000×g, 2 hours) and resuspended in PBS buffer. The virus was titrated as described previously [10], then inactivated by exposure to UV for 15 minutes. ELISA microplates were coated overnight at 37°C with 200 ng of viral proteins per well of inactivated semi-purified H5 or H7 virus diluted in PBS (pH 7.6), or with PBS only for one column as a blank. The wells were washed 3 times in PBS, and non-specific binding sites were blocked by a one-hour incubation at 37°C with gelatin (15 mg/ml in PBS). The plates were then washed 3 times in PBS containing 0.1% Tween 20 (PBS-Tween). Serum samples were pre-diluted to 1∶100 before, and then subjected to serial 2-fold dilutions that were distributed on the ELISA plate. Positive and negative serum standards were included on each plate as controls: five sera from naïve ducks or from naïve chickens were pooled and aliquoted to provide a standard negative serum control. After a one-hour incubation at 37°C, the plates were washed 4 times in PBS–Tween, and 100 µl of a 1∶200 dilution of either goat anti-duck (KPL, MD, USA) or anti-chicken (Sigma Aldrich, MO, USA) IgG serum conjugated to alkaline phosphatase in PBS–Tween was added in each well and incubated for 1 hr at 37°C. After 4 washes in PBS–Tween and one in PBS only, disodium *p*-nitrophenyl phosphate (100 µl/well) at a concentration of 1 mg/ml in 10% diethanolamine (pH = 9.8) was finally used as substrate. After 16 min at room temperature in the dark, the enzymatic reaction was stopped by adding 50 µl/well of 2 N NaOH, and absorbance was read using a spectrophotometer (Awarnesstech, FL, USA) at a wavelength of 405 nm. The serum sample titer was expressed as the reciprocal of the highest dilution for which the absorbance was at least 3 times the absorbance of the negative serum standard. Titers >20 were considered positive.

Hemagglutination Inhibition assays (HI), specific for H5 or H7, were performed following standard laboratory procedures, using horse red blood cells [12]. Titers >20 were considered positive.

### Virus titration by Real-time reverse transcription-PCR (qRT-PCR)

Cloacal and oropharyngeal swabs were collected and stored at −80°C until testing was performed. After thawing, swabs were soaked in 500 µL PBS. Viral RNA was extracted and purified from 150 µl of swabs sample fluid using viral RNA Isolation Nucleospin kit (Macherey-Nagel) following the manufacturer's instructions.

One-step RT-PCR was performed using Applied Biosystems PRISM 7000 Sequence Detection system (Quantitect SYBR green one-step real-time RT-PCR assays, QIAGEN) targeting the influenza A virus M gene [13]. A 25 µl RT-PCR mix consisted of 18 µL RT-PCR mix and 7 µl of purified RNA. Primers were used at a final concentration of 0.4 µM. Reverse transcription was carried out at 50°C for 30 min, followed by an activation of polymerase at 95°C for 15 min and by an initial denaturation step at 95°C for 15 seconds. cDNA was then amplified with 45 cycles of 95°C held for 15 seconds, 55°C for 30 seconds, and 72°C for 30 seconds. Fluorescence data were acquired at the end of each cycle in a single step.

Analysis of the melting curves was performed to assess the specificity of the PCR. For quantitation of virus shedding, a purified plasmid containing influenza M gene was used as standard. Serial tenfold dilutions of the plasmid were used to generate the standard curve. The quantitative PCR efficiency (slope of the standard curve) and linearity (*R* ^2^ value) for the serially diluted standard prepared was of consistently good quality.

### Data Analysis

Results were expressed as mean +/− standard error of the mean (SEM). Statistical difference between groups was assessed using the Mann-Withney U test.

## Results

### H5N3 primo-infection in ducks and chickens

**Figure 2:**
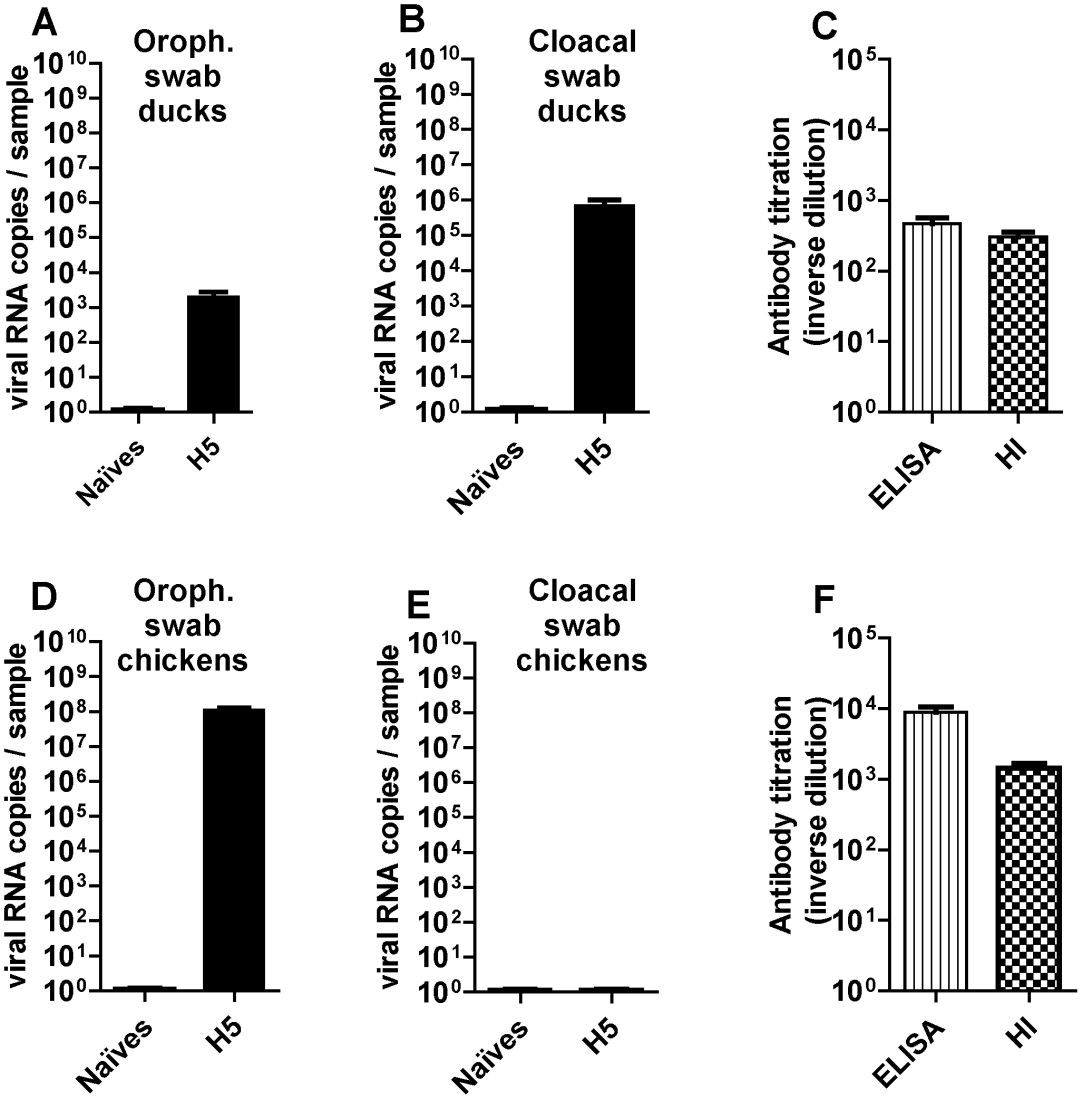
Viral excretion in upper airways (left) or in lower digestive tract (middle) and antibody response (right) to LPAIV H5N3 in ducks (top) or in chickens (bottom). Viral load in oropharyngeal swabs (A, D) and cloacal swabs (B, E) was expressed as viral RNA copies per sample and compared between control (naïves) and challenged birds two days (for chickens, corresponding to the peak of infection in lung) or three days (for ducks, corresponding to the peak of infection in cloacum) after inoculation. All the swabs were eluted in 1.5 ml PBS and were analysed using strictly the same protocol for RNA extraction and RT-PCR. Antibody titration (C, F) was expressed as inverse dilution of serum used for measurement by ELISA (vertical scores) or haemagglutination inhibition method (HI, draught-board).

In ducks, all the birds that were primo-infected at 3 weeks of age with the H5 virus remained clinically healthy during the 3 following weeks. At 3 days p.i., up to 10^4^ copies of viral RNA per sample were detected by real-time RT-PCR in the oropharyngeal samples, as compared to up to 10^6^ copies in the cloacal samples (Figures 2A and 2B, respectively). Twenty-one days after infection, viral loads were below the detection threshold of 100 copies per sample in most of the cloacal swabs (12/15), while no viral RNA was detected in the oropharyngeal swabs (data not shown). H5-specific ELISA and HI tests, that were performed at 2 weeks after inoculation showed a seroconversion in all infected ducks (Figure 2C).

In chickens, all birds also remained clinically healthy, although much higher titers of viral RNAs were measured in the tracheal swabs at 3 days p.i. (up to 10^8^ copies per sample; Figure 2D), in comparison to ducks (Figure 2A). Contrary to ducks, chickens did not excrete any virus by the fecal route (Figure 2E). Seroconversion of infected birds was observed (Figure 2F) at a higher level in chickens than in ducks.

### Homologous re-infection (H5N3/H5N3) in ducks and chickens

Three weeks after primo-infection with the H5 virus, ducks or chickens were reinfected with the same H5 virus (H5H5 groups) using the same oropharyngeal route, while at the same time 6 week-old naïve birds were inoculated for the first time with the H5 virus (H5 groups).

**Figure 3:**
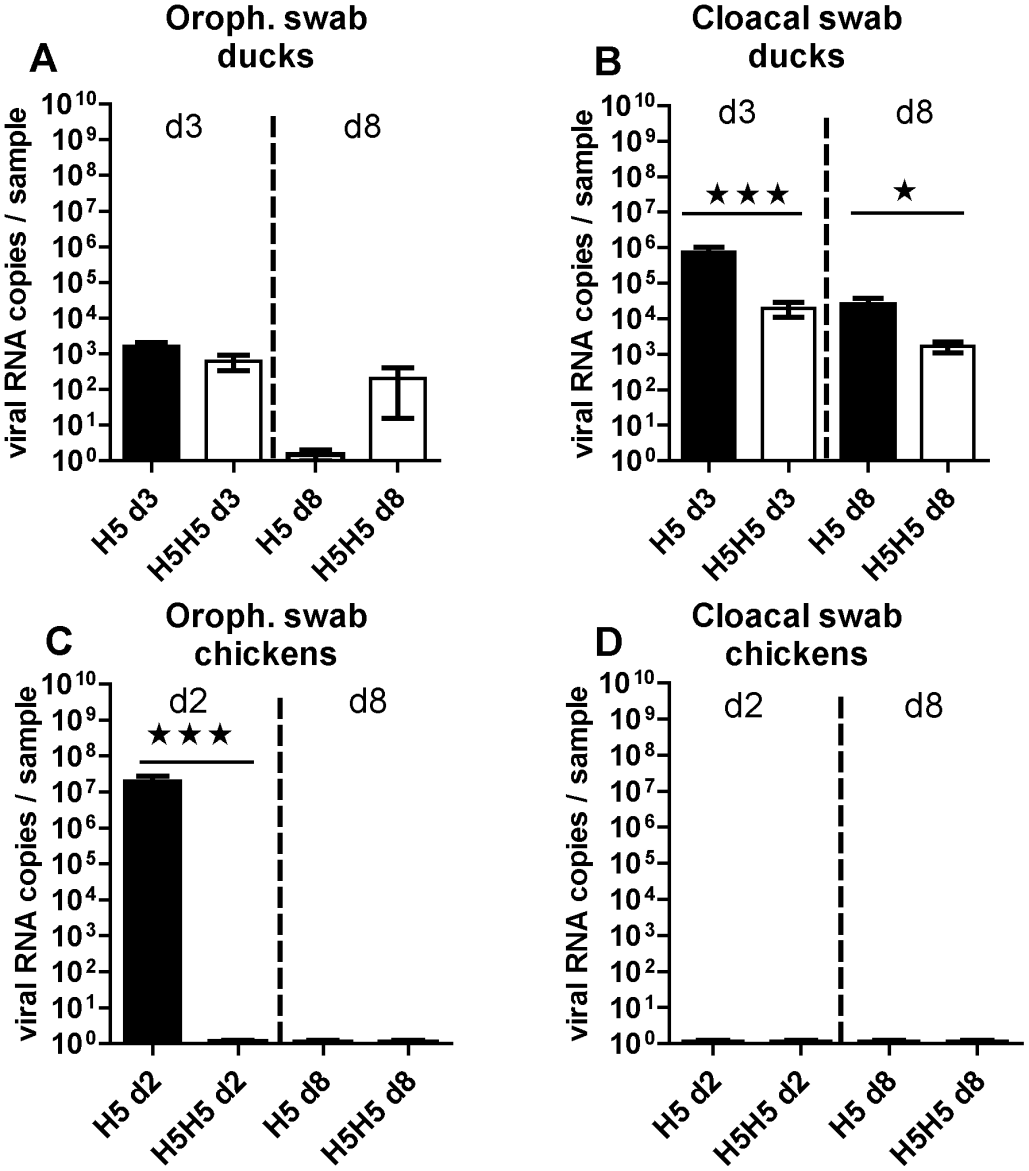
Viral excretion in upper airways (left) or in lower digestive tract (right) of LPAIV H5N3 in ducks (top) or in chickens (bottom). Birds were either primo-infected at six weeks of age (H5 groups) or re-infected at six weeks of age with the same virus as that used three weeks before (H5H5 groups). Viral titrations were measured at 2 days (d2, for chickens), 3 days (d3, for ducks) or 8 days p.i. (d8, for both bird species) and were expressed as viral RNA copies per sample and compared between primo-infected (H5 groups, black bars) and re-infected birds (H5H5 groups, white bars). All the swabs were eluted in 1.5 ml PBS and were analysed using strictly the same protocol for RNA extraction and RT-PCR. Significant differences between groups are indicated with asterisks (*, P<0.05; **: P<0.01; ***: P<0.001).

At 3 days p.i., all the ducks from the control H5 group showed significant levels of viral excretion, with up to 10^3^ and up to 10^6^ copies of viral RNA in the oropharyngeal and cloacal swabs respectively (Figure 3A and 3B). These titers are similar to those that were recorded at 3 days p.i. after the primo-inoculation at 3 weeks of age (compare Fig. 3A and 3B with Fig. 2A & 2B, respectively). A light gradual reduction of the viral load after one week post primo-infection could be detected. In addition, viral excretion was maintained only through the intestinal route at day 8 p.i. (compare the H5d8 groups in Fig. 3A and 3B), with a viral load of up to 10^5^ copies in the cloacal swabs while no viral RNA could be detected in the oropharyngeal samples (Figure 3A, H5 d8 group). Surprisingly, prior primo-infection with the H5N3 virus did not completely prevent viral excretion through neither oral nor cloacal route at 3 and 8 days after re-infection (Figure 3A and 3B, H5H5 group). However, when compared to primo-infected birds, the excretion was reduced by 100 times in the cloacal swabs at 3 days post-reinfection (compare H5 d3 with H5H5 d3 in Fig. 3B) while unaltered in oropharyngeal swabs (compare H5 d3 with H5H5 d3 in Fig. 3A).

In contrast with ducks, 6-wk old chickens that were primo-infected with the H5 virus excreted high amounts of virus though their upper respiratory airways at two days p.i. (Fig. 3C, left column). Again, these viral titers are similar to those recorded in the animals that were primo-infected at 3 weeks of age (compare with Fig. 2D). No virus could be detected at 8 days p.i., nor at any time point in the cloacal swabs (Figures 3C and 3D). Interestingly, early detection of the virus was completely inhibited in the oropharyngeal cavities of chickens that had been primo-infected with the same virus at 3 weeks of age (compare the H5 d2 and H5H5 d2 columns in Figure 3C). Taken together, these results suggest that the H5N3 primo-infection (i) *partially* inhibited the detection of H5 virus in the intestinal tract of ducks after a homologous re-infection, but (ii) *totally* prevented the replication of the homologous virus in the upper respiratory tract of chickens, as judged by the viral load detected in the oropharyngeal swabs.

**Figure 4:**
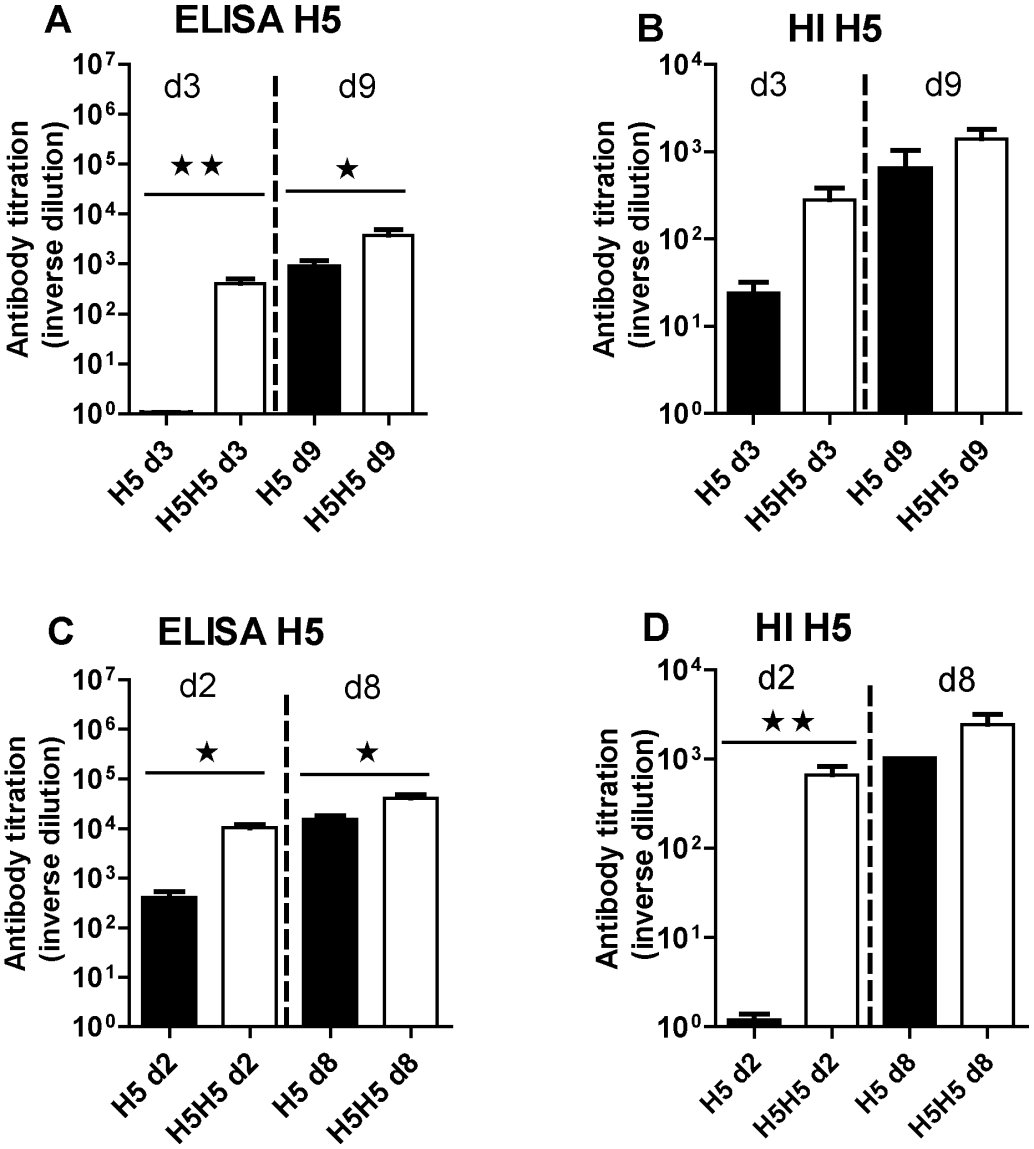
Antibody response to the H5N3 virus in ducks (top) or in chickens (bottom). Antibody titers were measured by ELISA (left) or HI method (right) from serum collected at the indicated time in days (d) after primary infection (H5 groups, black bars) or secondary infection (H5H5 groups, white bars) and were expressed as the reciprocal of the dilution of serum used for measurement as indicated in the Materials and Methods section. Significant differences between groups are indicated with asterisks (*, P<0.05; **: P<0.01; ***: P<0.001).

The antibody response against H5N3 was detectable as early as few days p.i. and increased with time after one week of infection when measured by ELISA or HI in both ducks (Figure 4A and 4B, respectively) and chickens (Figure 4C and 4D, respectively). However, the magnitude of this antibody response quantified by ELISA was always at least ten fold higher in chickens than in ducks (compare Fig. 4A and 4C). Interestingly, we observed that birds subjected to the homologous reinfection had increased antibody titers relative to the primo-infected birds (compare the H5 and H5H5 groups in Figs. 4A–D), reminiscent of a boost effect of the homologous reinfection on the humoral immune response that was observed in both bird species. This boost effect was more pronounced in the first days after re-infection and leveled off after one week.

### Heterologous reinfection (H5N3/H7N2) in ducks and chickens

**Figure 5:**
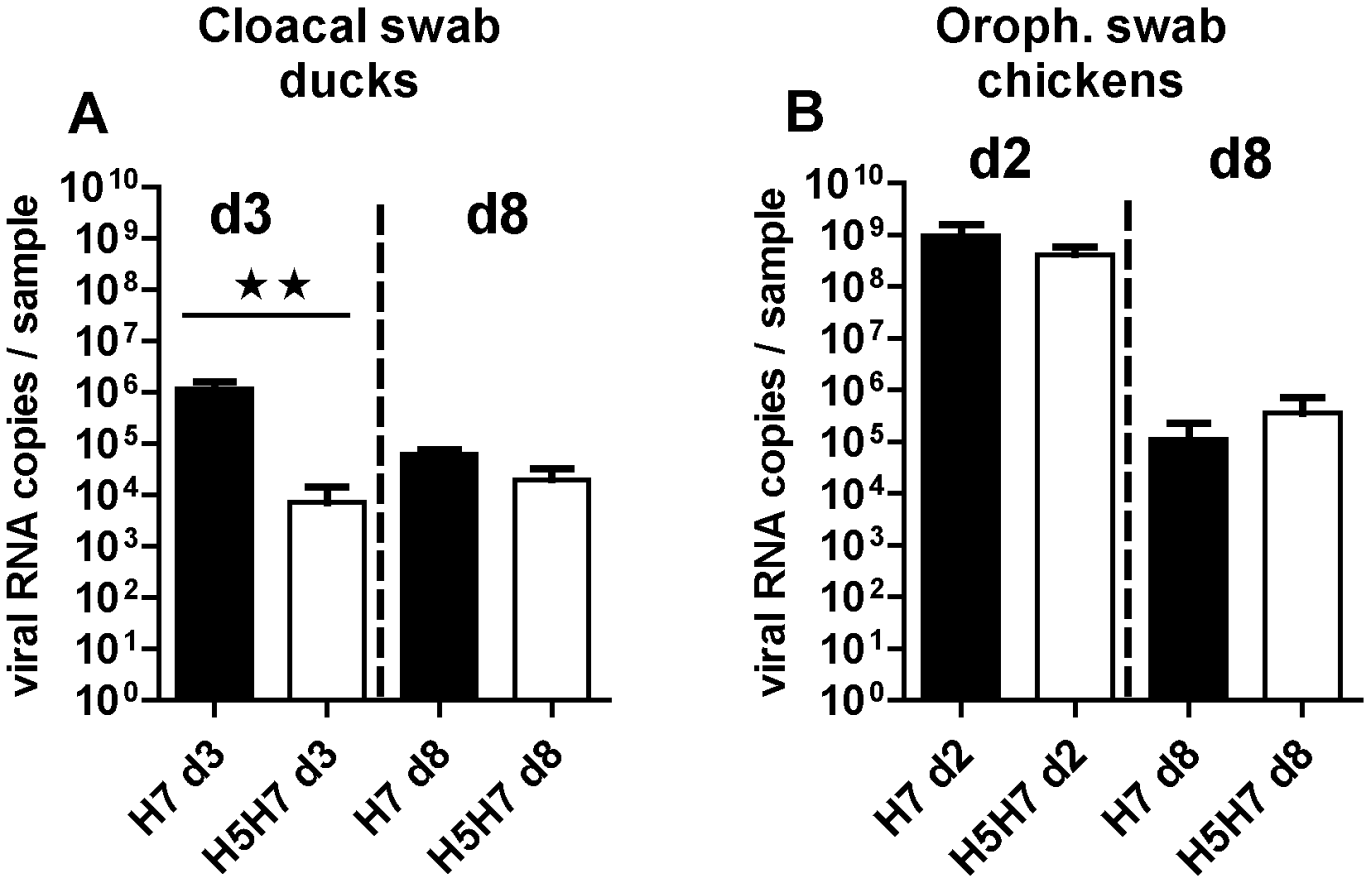
Viral excretion in lower digestive tract of ducks (A) or in upper airways of chickens (B) of LPAIV H7N2. Birds were either primo-infected at six weeks of age (H7 groups) or re-infected at six weeks of age with H7N2 virus, three weeks after H5N3 first inoculation (H5H7 groups). Viral titrations were measured 2 days (d2, for chickens) in oropharyngeal swabs, 3 days (d3, for ducks) in cloacal swabs, or 8 days (d8, for both birds species in their respective swabs) and were expressed as viral RNA copies per sample and compared between primo-infected (H7 groups, black bars) and re-infected birds (H5H7 groups, white bars). All the swabs were eluted in 1.5 ml PBS and were analysed using strictly the same protocol for RNA extraction and RT-PCR. Significant differences between groups are indicated with asterisks (*, P<0.05; **: P<0.01; ***: P<0.001).

Three weeks after primo-infection with the H5 virus, ducks or chickens were reinfected with the H7 virus (H5H7 group), while at the same time 6-week-old naïve birds were primo-inoculated with the same H7 virus (H7 group). Taking into account the results described above showing the different tropism of LPAIV for the duck digestive tract and the chicken respiratory tract, the results of viral load obtained after H7N2 infection were presented for each bird species according to the respective main target organ (Figure 5A and 5B). Indeed for both groups of ducks, cloacal excretion (Figure 5A) of the H7 virus was much more pronounced than oropharyngeal excretion (data not shown). At 3 days p.i., all the ducks that were primo-infected with the H7 virus excreted up to 10^6^ viral copies in the feces (Figure 5A, H7 d3 group). However, the viral titer was overall reduced by about 100 times in the ducks that had been previously primo-infected with the H5 virus (Figure 5A, H5H7 d3 group). At 8 days p.i., ducks still excreted up to 10^5^ of viral RNA copies per sample (H5H7 d8 group), whether or not they had been previously primo-inoculated (columns H7 d8 and H5H7 d8 in Fig. 5A). These results thus show that ducks that had been primo-infected with H5N3 were partially protected against H7N2 infection.

In contrast to ducks, H7N2 primo-infection of chickens induced no excretion of the virus in the feces (data not shown). However, we observed a very high level of excretion in the oropharyngeal swabs (up to 10^9^ viral RNA copies per sample) at 2 days post-infection (Figure 5B), coupled with symptoms of respiratory distress (data not shown). At 8 days post-inoculation, the viral load was greatly reduced by about 4 log in the upper respiratory tract of chickens. However, and in contrast to the situation of homologous reinfection in chickens, the primo-infection with H5N3 did not abrogate the H7N2 viral excretion in chickens (Figure 5B).

**Figure 6:**
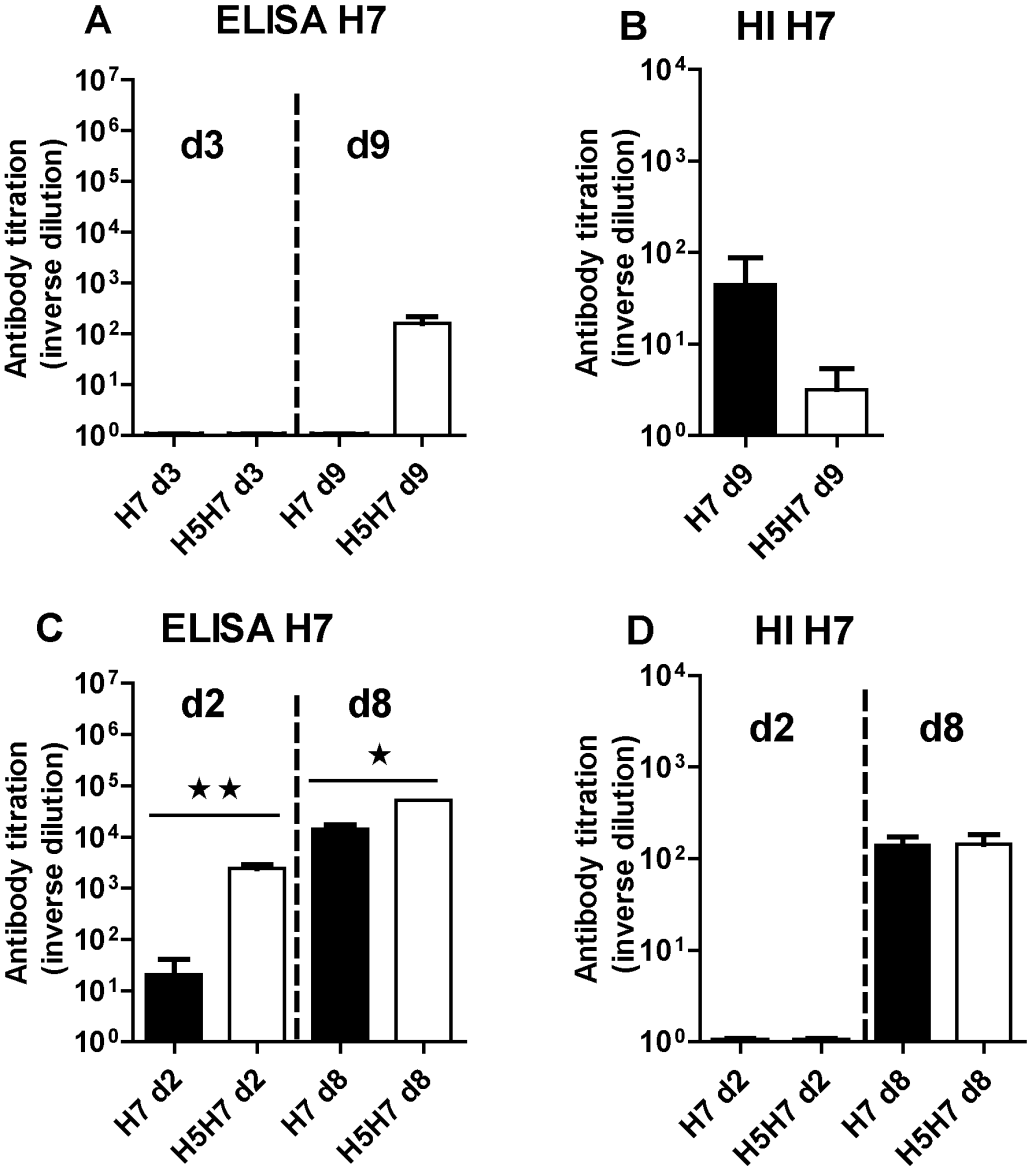
Antibody response to LPAIV H7N2 in ducks (top) or in chickens (bottom). Antibody titers were measured by ELISA (left) or HI method (right) from serum collected at the indicated time in days (d) after primary infection (H7 groups, black bars) or heterologous secondary infection (H5H7 groups, white bars) and were expressed as the reciprocal of the dilution used for measurement as indicated in the Materials and Methods section. Significant differences between groups are indicated with asterisks (*, P<0.05; **: P<0.01; ***: P<0.001).

No H7N2-specific antibodies were detectable in ducks by ELISA (Figure 6A) or HI (Figure 6B), except at day 9 post-inoculation and only at a low level. In chickens, an antibody response against H7N2 was observed at 2 and 8 days post-inoculation in the ELISA test and primo-infection with H5N3 boosted the response both at 2 and 8 days post-inoculation (Figure 6C). However the HI assay detected a H7N2-specific antibody response only at day 8 post-inoculation, with no effect of the prior primo-infection with the H5N3 virus (Figure 6D).

## Discussion

Different studies have previously analyzed, either in the chicken or in the duck, the effect of pre-exposure with LPAI virus on the outcome of a homo- or heterosubtypic infection with avian influenza [6], [7], [8]. Here, the influence of pre-exposure to a LPAI virus on the outcome of a homo- or heterosubtypic reinfection with LPAI viruses is analyzed through a comparative assessment in chickens and ducks of virus tropism and host antibody responses using the same experimental design.

In ducks, virus was detected mostly in the digestive tract for both the H5 and H7 viruses, whether or not animals had been previously primo-inoculated with the H5 virus. In chickens, the infection with both the H5N3 and H7N2 viruses was confined to the respiratory tract. The results obtained thus support the preferential tropism of the LPAI viruses, which is different between the two bird species: consistent with the literature data, viral replication preferentially takes place in the digestive tract of ducks and in the respiratory tract of chickens [2].

Noteworthy, the H5 virus was able to induce partial protection of ducks against the heterologous virus H7N2, but the level of protection was also partial against the homologous virus. This was accompanied by a moderate antibody response. These data are in agreement with previous studies performed in mallard ducks [3], [4] or when cross-immunity between H3N8 and H4N6 was compared in mallards, quails and pheasants [14]. The mechanisms sustaining this protection and the duration of this protective immunity still need to be elucidated. However, antibody response efficiency in ducks is not only limited quantitatively but may also be restricted functionally because of the truncated form of IgY missing the Fc fragment [15].

Primo-infection of chickens with the H5N3 virus totally prevented viral excretion after homologous reinfection, but had no effect when the reinfection was heterologous. This was highly correlated with the magnitude of chicken antibody response, in agreement with the data obtained by Sasaki et al. [16], and the lack of cross protection may be explained by the loss of affinity of the antibodies induced by the first virus towards the hetero-subtypic antigen. The protection reported here against the homo-subtypic virus and not against the hetero-subtypic one is in agreement with the homo-subtypic protection recently reported by Vergara-Alert et al. [7], even if the H7/H5 viral priming/challenge system they used (intranasal inoculation only and two HPAIV challenges used) was somewhat different.

Both bird species are able to control a LPAI primo-infection following activation of different arms of the innate immune response. However a recent genome-wide study indicates variations of immune-related genes both in terms of repertoire and of regulation by avian influenza virus infection between the two birds' species [17]. Specifically, chickens and ducks exhibit distinct TLR and type I interferon responses to LPAIV [18], [19]. This may be related to different players of the signalization cascade. RIG-I, which plays an important role in protection against influenza infection in mammals, is activated in ducks following influenza infection [19]. This intracytoplasmic molecular pattern sensor is not present in chickens [20], however LPAIV can still provoke a robust IFN-I response that is initiated by the RIG-like receptor MDA5 [21], [22]. In addition to the differences in the innate immune response, the resolution of infection to LPAI may also result from differences in the preferential replication sites, intestinal or respiratory according to the bird species. In birds nothing is known yet on the effect of the pattern of this innate immune response on the settlement of the specific immunity.

In both bird species, pre-existing immunity against H5N3 LPAI virus modified the outcome of an experimental challenge infection with the homologous LPAI virus. Interestingly, previous exposure to a hetero-subtypic LPAI virus may prevent viral shedding in ducks, while no protection could be observed in chicken.

Serology analysis indicates a boost effect of the H5N3-specific antibody response in ducks after homologous reinfection. Furthermore, the specificity of the antibody response seems to be most important in chickens, which could explain the total protection in the homologous challenge and the absence of protection in the heterologous one. Neutralizing antibodies against avian influenza virus infections are mainly directed against the variable regions of the HA and neuraminidase proteins and correlate with protective immunity against influenza strains of the same subtype. These specific antibodies do not offer protection against viruses of distinct HA or NA subtypes. While the immunity to influenza is primarily subtype-specific, epidemiological evidence suggests the existence of heterosubtypic cross-immunity. However, the immune response involved in the heterosubtypic protection is not fully characterized. It is now widely accepted that the cell-mediated cytotoxic immune response against conserved antigen targets is a key pathway of cross-subtype immunity [23]. In contrast, there are some data demonstrating a role for antibodies in heterosubtypic immunity in mammals [24]. In birds, Berhane et al. showed some degree of cross-protection to a H5N1 challenge in Canada geese that were previously infected with a H3N8 LPAI virus [25]. Further evidence of heterosubtypic immunity in wild ducks has been provided very recently by individual follow-up of mallards through capture and recapture studies, suggesting multiple infections of the same birds by distinct subtypes of AIV [26]. The mechanism responsible for the survival of some of the H3N8 pre-exposed birds was roughly correlated with the antibody response against nucleoprotein of influenza A viruses [25]. A role for macrophages in heterosubtypic immunity is also supported by the study of Sambhara et al. [27]. Alternatively, cross-protective antibodies may work in conjunction with NK cells as demonstrated for protection of mice by M2-specific antibodies [28].

Further studies are needed to better understand the differences observed between chickens and ducks during influenza infection. These differences may be associated with different main sites of viral replication, or with differences in maturation of the immune system, though the functional ontogeny of duck's immune system seems to share major features with that of chicken [29]. Dissection of the distinctive traits of the innate and adaptive immune pathways between waterfowl and gallinaceous birds is a priority and will be greatly supported by the recent achievements in the mapping of duck's genome [17]. The mechanisms underlying these differences of the level and duration of protection are worth being clarified for a better understanding of both chicken sensitivity to avian influenza and the “reservoir” status of waterfowl.
